# Anisotropy Measure from Three Diffusion-Encoding Gradient Directions

**DOI:** 10.1016/j.mri.2022.01.014

**Published:** 2022-02-02

**Authors:** Santiago Aja-Fernández, Guillem París, Carmen Martín-Martín, Derek K. Jones, Antonio Tristán-Vega

**Affiliations:** LPI, ETSI Telecomunicación, Universidad de Valladolid, Castilla y León, Spain; LPI, ETSI Telecomunicación, Universidad de Valladolid, Castilla y León, Spain; LPI, ETSI Telecomunicación, Universidad de Valladolid, Castilla y León, Spain; Cardiff University Brain Research Imaging Centre (CUBRIC), School of Psychology, Cardiff University, UK; LPI, ETSI Industriales, Universidad de Valladolid, Castilla y León, Spain

**Keywords:** Diffusion MRI, fractional anisotropy, diffusion anisotropy, fast acquisition, white matter

## Abstract

We propose a method that can provide information about the anisotropy and orientation of diffusion in the brain from only 3 orthogonal gradient directions without imposing additional assumptions. The method is based on the Diffusion Anisotropy (DiA) that measures the distance from a diffusion signal to its isotropic equivalent. The original formulation based on a Spherical Harmonics basis allows to go down to only 3 orthogonal directions in order to estimate the measure. In addition, an alternative simplification and a color-coding representation are also proposed. Acquisitions from a publicly available database are used to test the viability of the proposal. The DiA succeeded in providing anisotropy information from the white matter using only 3 diffusion-encoding directions. The price to pay for such reduced acquisition is an increment in the variability of the data and a subestimation of the metric on those tracts not aligned with the acquired directions. Nevertheless, the calculation of anisotropy information from DMRI is feasible using fewer than 6 gradient directions by using DiA. The method is totally compatible with existing acquisition protocols, and it may provide complementary information about orientation in fast diffusion acquisitions.

## 1 Introduction

The term Diffusion Magnetic Resonance Imaging (DMRI) refers to a set of diverse imaging techniques that, applied to brain studies, provide useful information about the organization and connectivity of the white matter. The most relevant feature of DMRI is its ability to measure orientational variance in the different tissues, i.e., anisotropy, a feature that is mostly used in research. In the clinical practice, and as a complement to structural studies, there are protocols that incorporate a fast acquisition to obtain a measure of the *amount* of diffusion. A common implementation in commercial scanners, like EPI-DWI, acquires only 3 separate orthogonal diffusion weighted images (DWIs) with diffusion gradients aligned with directions (*x, y, z*). These 3 DWIs are averaged into a final combined image [1] that resembles measures like the Mean Diffusivity (MD) [2] or the Average Sample Diffusion (ASD) [3]. Due to the limitation in the number of gradient directions, no extra information is provided. If a measure of anisotropy and orientation of the diffusion wants to be extracted, there is a minimum requirement of 6 acquired DWIs in order to estimate the components of the diffusion tensor (DT) [4]. Under the DT approach, it would still be possible to calculate an anisotropy measure with fewer than 6 gradient directions, but we must impose a restricted model that reduces the number of values to estimate, like, for instance, assuming that the diffusion has a cylindrical symmetry. Nevertheless, regardless of the used methodology, it is well known the intrinsic inability of dMRI measures to properly characterize different spatial orientation with fewer than 6 gradient directions.

In this paper we propose a new method that can provide additional information about the anisotropy in the diffusion from only 3 orthogonal gradient directions. This method is totally compatible with existing fast diffusion acquisitions since it only makes use of the same 3 DWIs already acquired. This way, no extra scanning time is needed: the same sequence that provides MD images can also provide anisotropy information.

The method is based on a novel anisotropy metric called Diffusion Anisotropy (DiA) proposed in [5]. The metric measures the distance from the actual diffusion signal to its isotropic equivalent. Its original formulation relies on the fitting on the signal using a basis of Spherical Harmonics (SH), but an alternative simpler formulation is here proposed to be exclusively used with 3 orthogonal gradient directions. In addition, we also present a color-coding method, like the one used for the Fractional Anisotropy (FA) in DT imaging. We carry out some examples and tests to show that, although the variability of the anisotropy image is high (compared to the one calculated with more gradient-directions), it succeeds in providing structural information of the white matter with just 3 acquired directions for those tracts aligned with the axis.

Due to the limitations of DMRI, when working with fewer than 6 gradient directions, those tracts not aligned with the axis will be underestimated by the procedure. Thus, we must recall that this method is not initially intended to carry out clinical studies or to obtain detailed anisotropy information, but simply to complement existing acquisition methods with an anisotropy measure. The acquisition remains unchanged, only some extra processing is needed, and new information is then provided.

## 2 Methods

### 2.1 Diffusion Anisotropy

In [5], authors proposed a series of advanced anisotropy measures that could be calculated from a single shell acquisition. Among them, the Diffusion Anisotropy (DiA) was presented as a robust alternative to the FA. DiA assumes a mono-exponential diffusion profile for the normalized magnitude signal provided by the MRI scanner, *E*(**q**):

(1) $$E(q)\;=\;E(q,\;u)\;=\;\exp\;(-4\pi^{2}\tau q^{2}\;D(u))\;=\;\exp\;(-b\;\cdot \;D(u)).$$

*D*(**q**) = *D*(*q*, **u**) > 0 is the *diffusivity signal*, also known as the Apparent Diffusion Coefficient (ADC), *b* = 4*π*^2^*τ*‖**q**‖^2^ is the b-value, *τ* is the effective diffusion time, *q* = ‖**q**‖ and u∈S is a unit direction in space where ‖**u**‖ = 1 and **q** = *q***u**. Note the mono-exponential constraint translates in the diffusivity *D*(*q*, **u**) being independent on the radial direction: *D*(*q*, **u**) ≡ *D*(**u**)Under this assumption, the DiA is defined as [5]:

(2) $$\text{DiA=}\sqrt{1-\frac{{\left[\int_{S}D(u)du\right]}^{2}}{4\pi \cdot \int_{S}D^{2}(u)du}}.$$

The integration on the surface of the unit sphere, *S* = {**u** ∈ ℝ^3^ : ‖**u**‖ = 1}, from a limited number of samples can be performed by fitting corresponding signals in the basis of Spherical Harmonics (SH), whose 0-th order coefficient is defined as:

(3) $$C_{0,0}\{H(u)\}=\frac{1}{\sqrt{4\pi }}\int_{S}H(u)du.$$

This way, a practical implementation of DiA was originally defined as:

(4) $$\text{DiA=}\sqrt{1-\frac{C_{0,0}^{2}\{D(u)\}}{\sqrt{4\pi }\cdot C_{0,0}\{D^{2}(u)\}}}.$$

This implementation can be seen as a generalization of the Coefficient of Variation of the Diffusion (CVD), defined in [3], and an alternative definition to the Generalized Anisotropy (GA) proposed in [6]. Finally, note that, as mentioned in [7, 8], FA-like measures suffer from confounding factors derived from the MRI resolution being bigger than most of the hydrogen molecules displacement in brain tissues. Therefore, both FA and DiA are the result of an averaged measure of the diffusion contributions over the voxel being studied.

### 2.2 Simplified DiA and Color-by-orientation

The advantage of the definition of DiA using a SH base, like the one proposed in eq. (4), is that the integral can be roughly estimated from just 3 orthogonal values. Let *D_x_*(**x**), *D_y_*(**x**), and *D_z_*(**x**) be the diffusion signals acquired for these such directions (that, in principle, we assume aligned with the corresponding axes ‘x’, ‘y’, and ‘z’). We can calculate the *average diffusivity* by simply drawing:

(5) $${\text{D}}_{\text{AV}}(x)=\frac{D_{x}(x)+D_{y}(x)+D_{z}(x)}{3}.$$

At the same time, DiA can be calculated using eq. (4):

(6) $$\text{DiA(}x\text{)=}\sqrt{1-\frac{C_{0,0}^{2}\{D_{x}(x),\;D_{y}(x),\;D_{z}(x)\}}{\sqrt{4\pi }\cdot C_{0,0}\{D_{x}^{2}(x),\;D_{y}^{2}(x),\;D_{z}^{2}(x)\}}}.$$

However, since the three acquired DWIs are orthogonal and aligned with the Cartesian axes, the DiA can be alternatively calculated using a simplified formulation [3]:

(7) $$\text{DiA(}x\text{)=}\sqrt{1-\frac{{\left(D_{x}(x)+\;D_{y}(x)+\;D_{z}(x)\right)}^{2}}{3\cdot (D_{x}^{2}(x)+\;D_{y}^{2}(x)+\;D_{z}^{2}(x))}}.$$

Note that, in this case, since we are assuming three orthogonal vectors, we do not need to use the gradient directions in order to calculate the DiA.

We can also provide color-coded anisotropy information using DiA. In DT imaging, the anisotropy is usually coded using a RGB color system [9] in which blue is superior-inferior, red is left-right, and green is anterior-posterior. For visual purposes, the luminance of the color is weighted by the FA. Analogously, we define the RGB components as a function of the three orthogonal directions normalized by the average diffusivity, so that:

(8) $$\begin{array}{lll} \text{r}(x) & = & \text{DiA}(x)\times \frac{D_{x}(x)}{{\text{D}}_{\text{AV}}(x)} \end{array};$$

(9) $$\begin{array}{lll} \text{g}(x) & = & \text{DiA}(x)\times \frac{D_{y}(x)}{{\text{D}}_{\text{AV}}(x)} \end{array};$$

(10) $$\begin{array}{lll} \text{b}(x) & = & \text{DiA}(x)\times \frac{D_{z}(x)}{{\text{D}}_{\text{AV}}(x)} \end{array}.$$

Note that this formulation implicitly assumes that the three acquired gradient directions are orthogonal and they are aligned with the axis (*x, y, z*). Thus, the color coding must be interpreted as orientation of the structures with respect to the axis.

**Figure 1:**
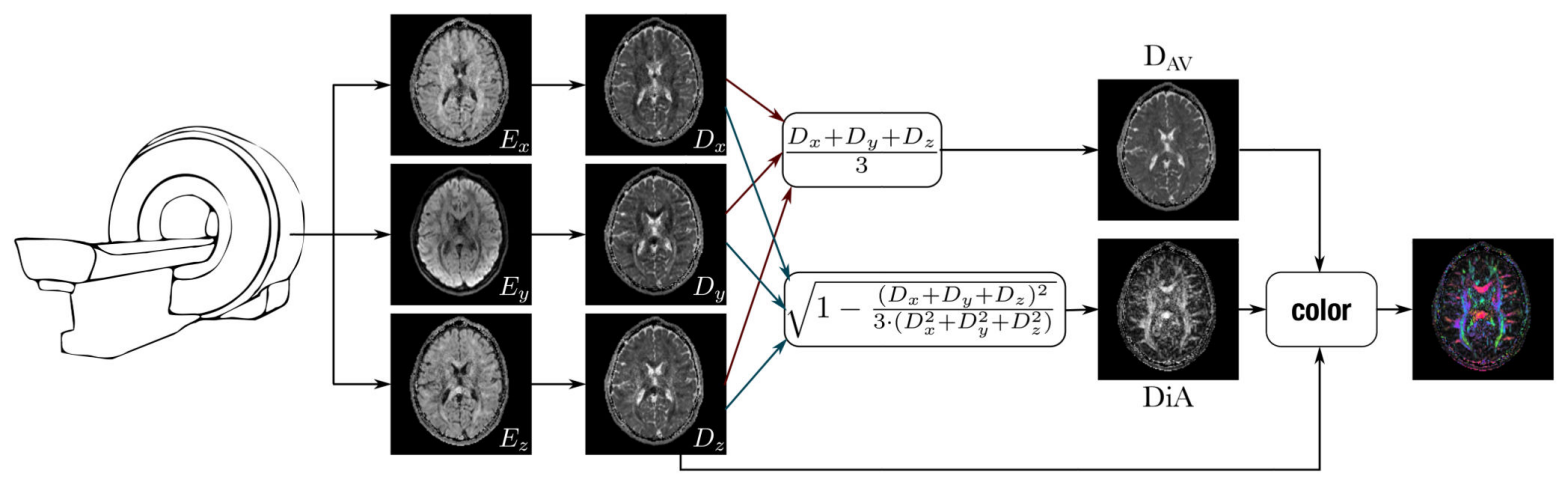
Scheme of the calculation of the different metrics derived from 3 DWIs acquired with 3 orthogonal gradient directions.

The calculation of the different metrics from the 3 acquired orthogonal measures is surveyed in Fig. 1.

## 3 Results

### 3.1 Data used for the experiments

An MRI volume (UVa) from a healthy control was acquired using a Philips Achieva 3T unit (Philips Healthcare, Best, The Netherlands) in the MRI facility at the Universidad de Valladolid (Valladolid, Spain). The acquisition was obtained with these parameters: TR = 9000 ms, TE = 86 ms, flip angle = 90^*o*^, one baseline volume, b-value = 1000 s/mm^2^, 128×128 matrix size, 2×2×2 mm^3^ of spatial resolution and 66 axial slices covering the whole brain. 3 different sets were considered: 3, 6 and 61 gradient directions. For the acquisition with 3 directions, the acquired gradients are aligned with the axis. Data were preprocessed using MRtrix software [10] for correction of eddy currents, motion, and field inhomogeneities.

In addition, the **Human Connectome Project (HCP)**^1^ database was also used, specifically volumes MGH1010 and MGH1016, acquired on a Siemens 3T Connectom scanner with 4 different shells at *b* =[1000, 3000, 5000, 10000] s/mm^2^, with [64, 64, 128, 256] gradient directions each, in-plane resolution 1.5 mm and slice thickness 1.5 mm. We will only make use of the innermost shell (*b* = 1000 s/mm^2^ and 64 gradient directions).

### 3.2 Visual Assessment

**Figure 2:**
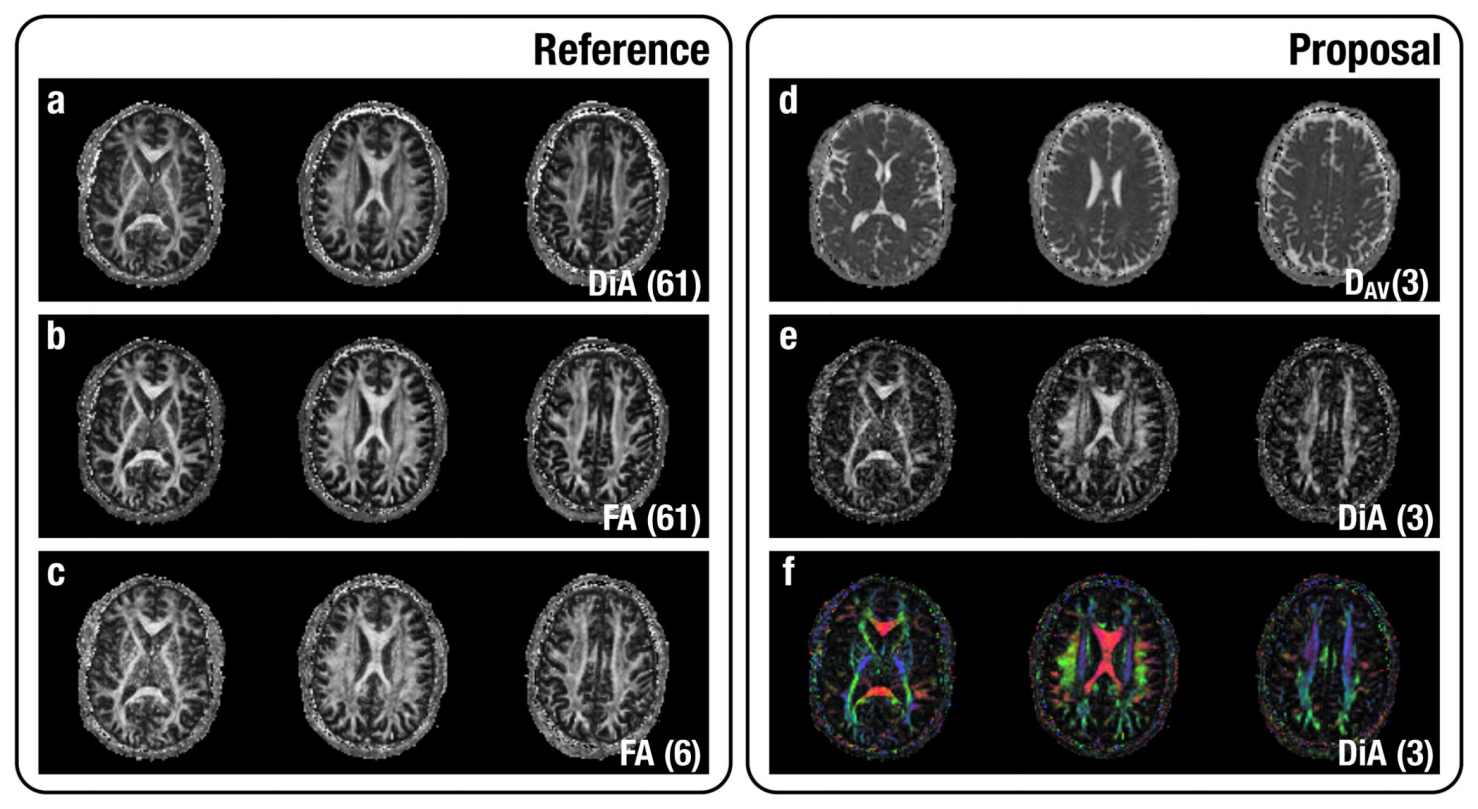
Visual assessment of proposed methods (2). Slices (31, 40, 55) from the UVa volume are shown. For the sake of comparison we have added (a) DiA (using 61 gradient directions); (b) FA (using 61 gradient directions; (c) FA (using 6 gradient directions. The proposed metrics are calculated with 3 gradient directions: (d) Average Diffusivity; (e) DiA; (f) DiA with orientation color code.

First, we calculate the proposed metric over 3 slices (31, 40 and 55) from the UVa volume. The DAV and DiA were calculated using only 3 DWIs from the shell at b=1000 s/mm^2^ using the simplified expression in eq. (7). For the sake of comparison, we have also calculated the FA at b=1000 s/mm^2^ with 61 and 6 gradient directions and DiA with 61 (UVa) directions. In the latter case, DiA was calculated using eq. (4); the SH are fitted with a Laplace-Beltrami penalty λ = 0.006. Results are shown in Fig. 2.

Although the visual quality of DiA calculated with 3 directions, Fig. 2-(e), is clearly poorer than FA and DiA with 61 directions (which is obvious), note that DiA succeeds in estimating information about orientation and anisotropy with just 3 gradient directions. Main structures are visible within the white matter, even clearer in the colored version, Fig. 2-(f). Thus, the same fast acquisition that can produce information about the amount of diffusion can also be used to provide rough information about the orientation of such diffusion. Note that the high quality of the images calculated with only 3 gradients is due to the large voxel size used (2 × 2 × 2 mm^3^), which assures a high SNR in the data.

### 3.3 Numerical assessment

**Figure 3:**
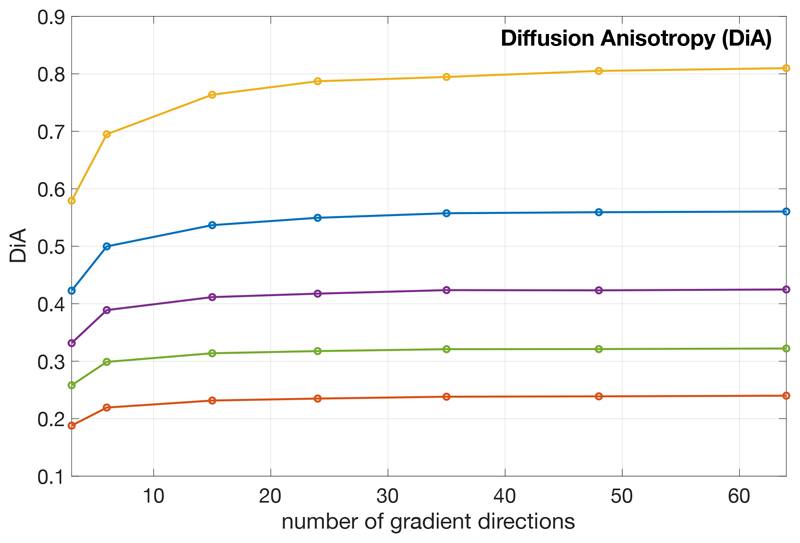
Evolution of DiA with the angular resolution (number of gradient directions), using data from HCP. The volume has been clustered in 6 different sets (for original DiA with 64 directions) and the median of each set is shown. Centroids of the data *C_L_* = {0.24, 0.32, 0.42, 0.57, 0.82}.

Next, we quantified the loss of information in DiA when calculated using only 3 different orientations. First, we tested the dependency of DiA on the number of diffusion samples taken in a given shell. To that end, we used a whole volume from the HCP data, MGH1016. The volume was divided in 6 different regions according to their diffusion features. The DiA was first calculated at b=1000 s/mm^2^ using 64 directions and those voxels with DiA < 0.1 removed. The remaining voxels were clustered in 6 different groups using k-means. Each voxel in the white matter was assigned to one cluster using its DiA value and the minimum distance. The following test was carried out: we began with the 64 samples (gradient directions) and uniformly downsampled this set to obtain either 3, 6, 15, 24, 35 and 48 diffusion directions subsets^2^. The DiA was computed for each considered case, and the median value inside each of the six clusters is depicted in Fig. 3. Although DiA shows it is a consistent measure when it is calculated using over 20 different orientations, for fewer gradient directions this measure is underestimated. This effect is much more noticeable when using only 3 directions. However, note that the separation between clusters remains constant. This suggest that the differences in the anisotropy detected by these measures can still be seen when using 3 orientations (at least in this example).

### 3.4 Variability of DiA with orientation

**Figure 4:**
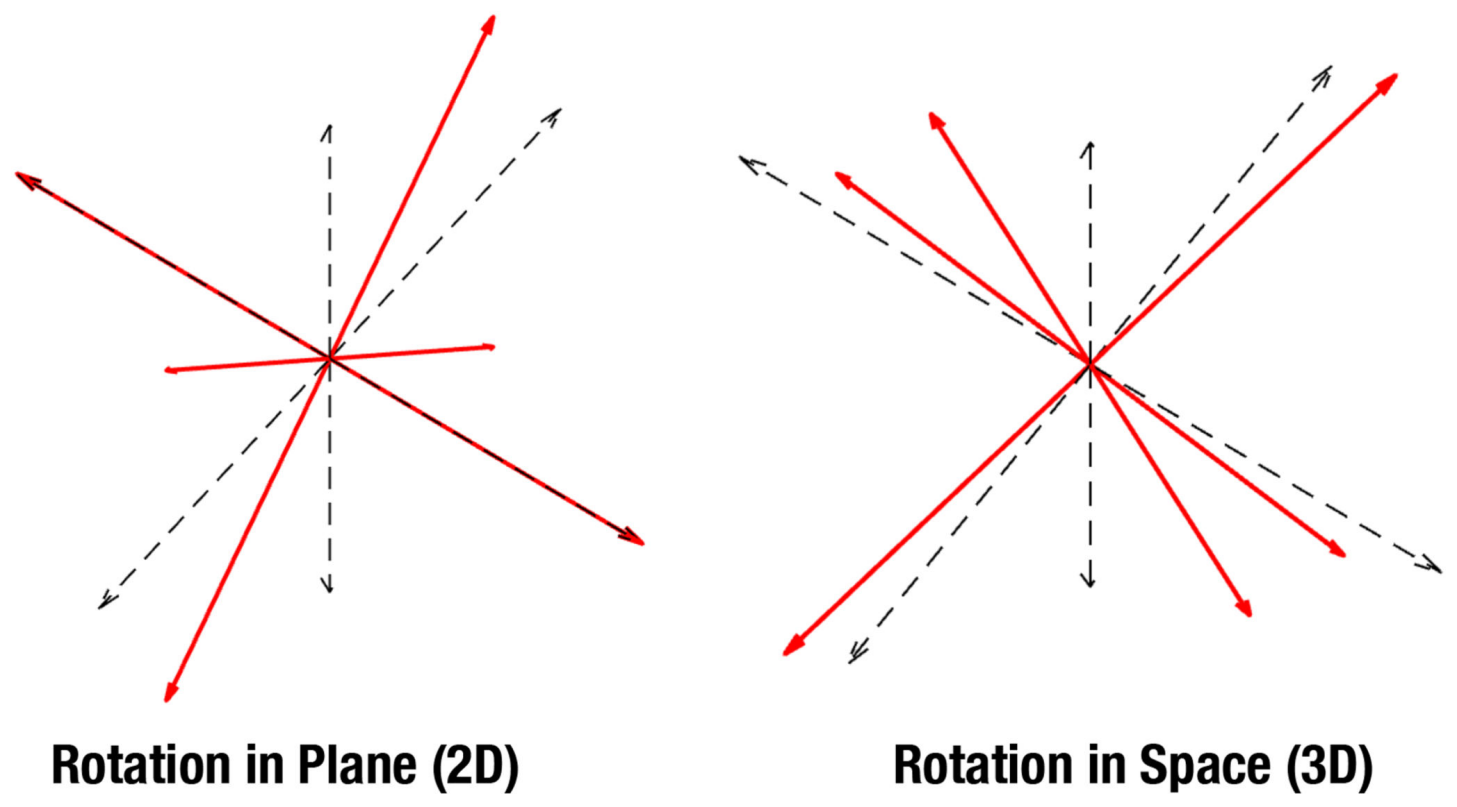
Directions of the eigenvectors of the synthetic tensor (red) in relation with the axis (black). Two different rotations are applied. LEFT: rotation on a plane. RIGHT: spatial rotation.

One of the issues with anisotropy measures in dMRI is the intrinsic inability to properly characterize fibers in all different spacial orientation when fewer than 6 gradient directions are considered. Thus, in order to quantify the capability of this method to identify fibers that are not aligned with the axis, we will carry out a simple simulation: we generate a synthetic tensor with eigenvalues [1, 0.3, 0.3] × 10^-3^ mm^2^/s totally aligned with axis. This corresponds to FA=0.6444. We rotate the tensor according to two different rotation schemes, see Fig. 4:

1. Rotation in plane, so one of the components of the tensor is aligned with one axis. The rotation matrix is:
  $$R_{M}=[\begin{array}{ccc} \cos(\theta ) & 0 & \sin(\theta )\\ 0 & 1 & 0\\ -\sin(\theta ) & 0 & \cos(\theta ) \end{array}]$$
2. Spatial rotation, so none of the components are aligned with any axes. The rotation matrix is:
  $$R_{M}=[\begin{array}{ccc} \frac{1}{3}+\frac{2}{3}\cos(\theta ) & \left(1-\cos(\theta ))\frac{1}{3}-\frac{1}{\sqrt{3}}\sin(\theta \right) & \left(1-\cos(\theta ))\frac{1}{3}+\frac{1}{\sqrt{3}}\sin(\theta \right)\\ \left(1-\cos(\theta ))\frac{1}{3}+\frac{1}{\sqrt{3}}\sin(\theta \right) & \frac{1}{3}+\frac{2}{3}\cos(\theta ) & \left(1-\cos(\theta ))\frac{1}{3}-\frac{1}{\sqrt{3}}\sin(\theta \right)\\ \left(1-\cos(\theta ))\frac{1}{3}-\frac{1}{\sqrt{3}}\sin(\theta \right) & \left(1-\cos(\theta ))\frac{1}{3}+\frac{1}{\sqrt{3}}\sin(\theta \right) & \frac{1}{3}+\cos(\theta )\frac{2}{3} \end{array}].$$

**Figure 5:**
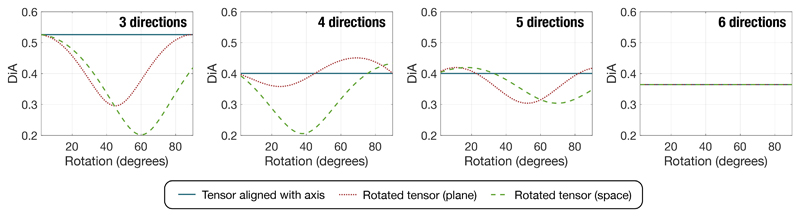
DiA calculated from 3 acquisitions. The main diffusion direction of the tensor is rotated a certain angle so that the fiber is not aligned with the axis.

The tensor is sampled using three to six directions. For the case of 3 directions, these correspond to the Cartesian axes. For the sake of simplicity, no noise or simulated artifacts are added to the tensor. The diffusivity *D*(**x**) is reconstructed and the DiA is calculated for each case. The values of DiA for the different sampling schemes and for different rotation angles are shown in Fig. 5 (plane rotation in red and spatial rotation in green).

Note that, according to the figure, when the DiA is estimated with only 3 directions, there is a clear underestimation of the metric when the main diffusion direction is not aligned with the directions of the acquired gradients. If we focus on the in plane rotation, the maximum error arises precisely for the 45^*o*^ angle, when the main direction is diagonal to the axis. This is also the case for 4 and 5 directions, while with 6 directions the same DiA is provided, regardless of the orientation of the tensor.

**Figure 6:**
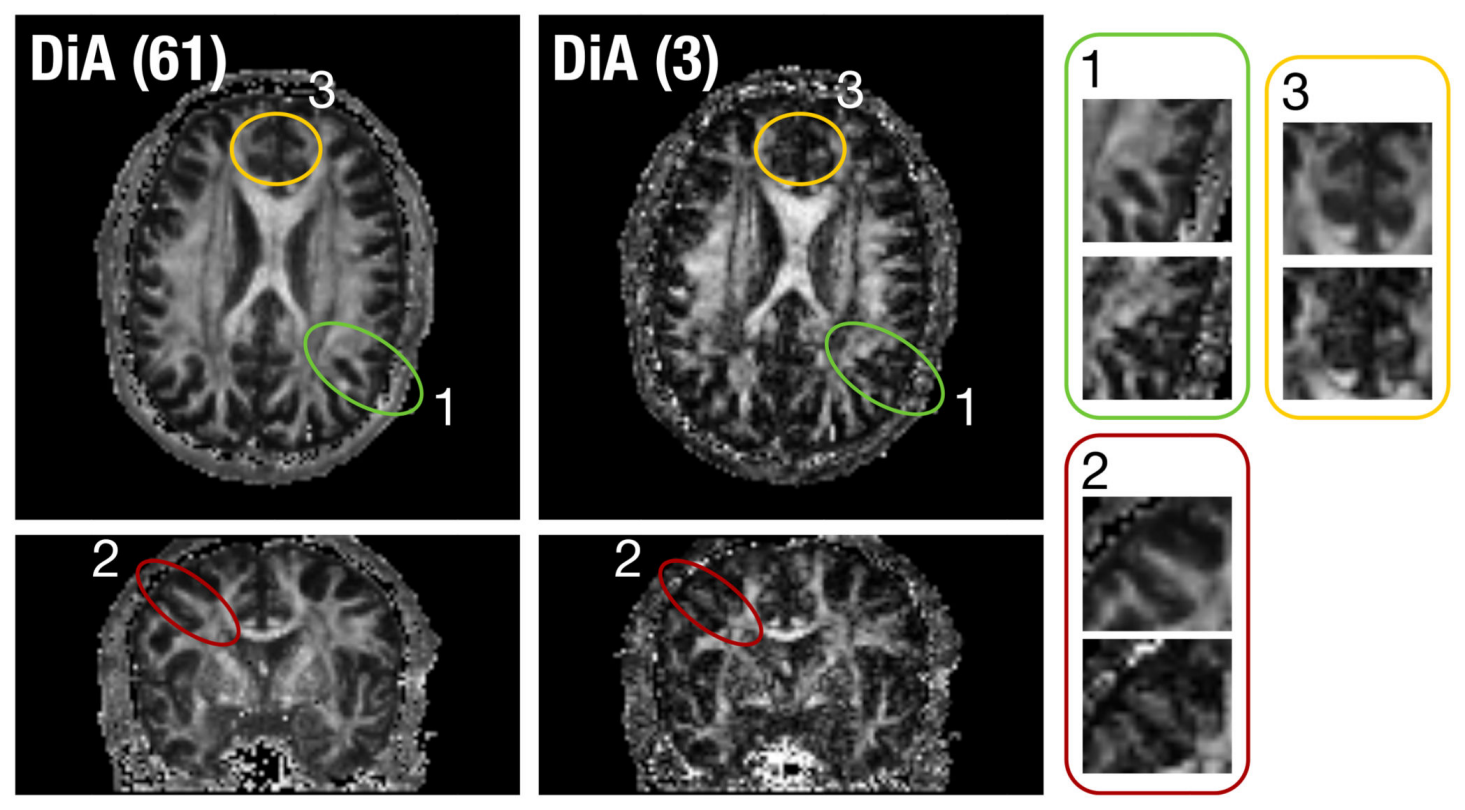
DiA calculated with 61 and 3 gradient directions: Comparison of the anisotropy in three different areas for bundles not aligned with the axis.

This experiment raises the main weakness of this method, the underestimation of the anisotropy of those fibers not aligned with the axis. This effect can also be seen on real data. In Fig. 6 we show one axial and one coronal slice from the UVa volume. We have calculated DiA for 61 and 3 gradient directions in order to compare the loss of information. We have highlighted some of the fiber bundles that are not aligned with the axis. Let us first focus on the structures circled in green and red (numbers 1 and 2). These structures would correspond to the rotation in plane in Fig. 5, with an angle of around 45^*o*^ respect to the axis. According to the previous experiment, the anisotropy here would experience its maximum underestimation. This is the case in Fig 6: the bundles in DiA with 3 gradient directions show a reduced value, when compared to the 61 case. However, in both cases (1 and 2), although reduced, the bundles are still present. A similar effect can be found on the area number 3. A small structure has almost disappeared due to its orientation.

**Figure 7:**
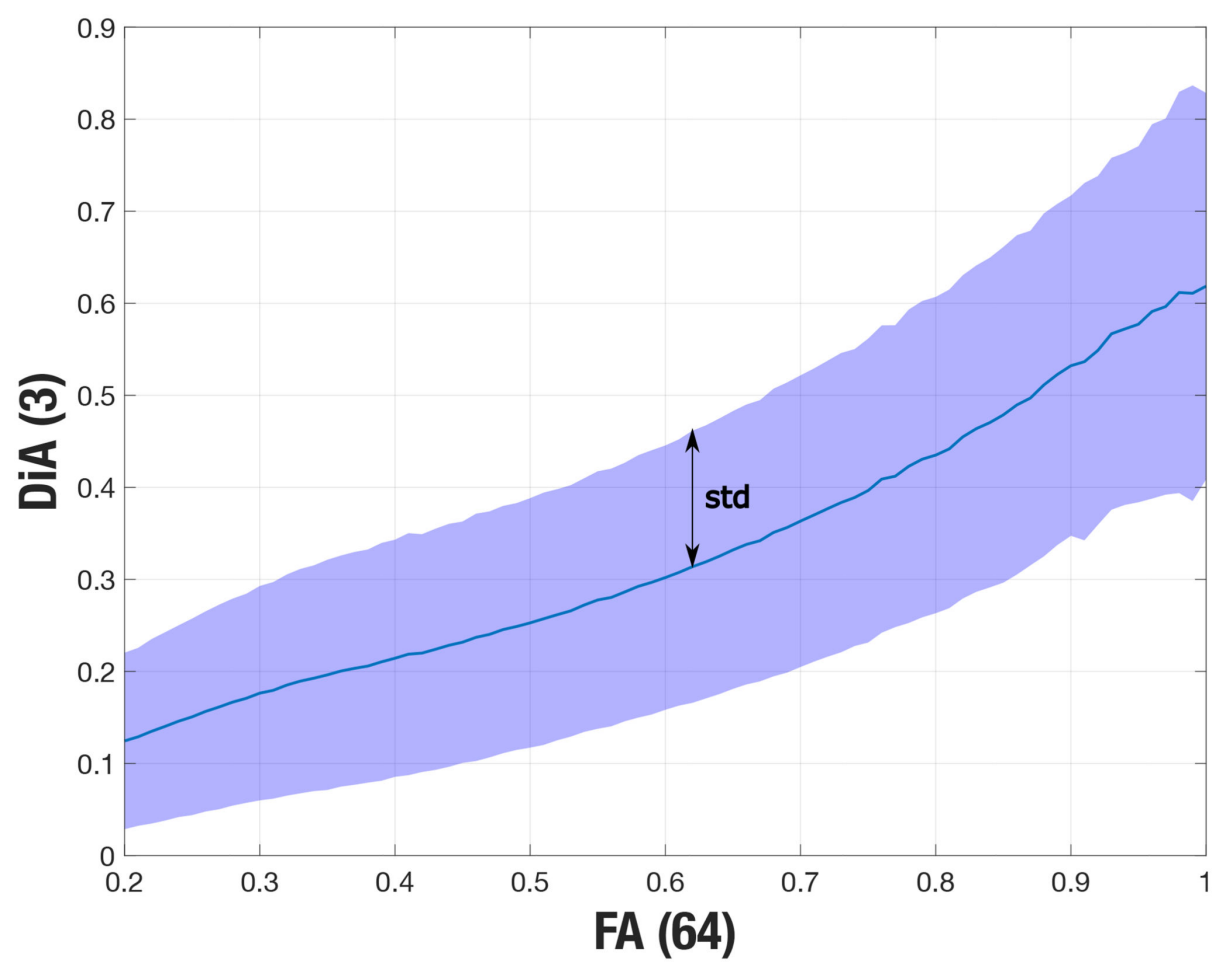
Variability of the DiA as a function of the orientation of the acquired direction. HCP data have been used. In blue, the median along different gradient configurations.

Finally, we numerically quantified this variability over the HCP data (volume MGH1010). We downsample the 64 acquired directions to sets of 3 directions through an exhaustive search with all possible orientations. This way, we consider sampling schemes not aligned with the axis and in all the possible orientations. DiA is calculated for each acquisition set using eq. (4). The median and standard deviation is calculated along the different configuration. Results as a function of the FA are shown in Fig. 7.

Despite its great variability with the orientation of the acquired gradients, DiA also shows a great correlation (in median) with the FA, revealing that the differences in the anisotropy detected by FA can still be seen when using DiA 3 orientations, although with a greater variance.

## 4 Discussion and Conclusions

The calculation of anisotropy measures over diffusion data is usually limited by the 6 gradient directions needed to estimate the components of the diffusion tensor. Hence, in those fast acquisitions for which only 3 orientations are acquired, only information about the *amount* of diffusion can be inferred. That is the case of fast diffusion sequences in commercial scanners (like EPI-DWI) in which the installed software produces an image which is the average of 3 images acquired with 3 orthogonal gradient directions where no information about the orientation of the diffusion is present.

In this work we have proposed a method that is able to calculate a rough anisotropy image that could give complementary information about the anisotropy and orientation of the diffusion with just those 3 orthogonal directions. In addition, we also provide a color-coded version that helps in better understanding the orientation of the different structures. The method is totally compatible with existing fast acquisition sequences, and it does not require extra data: the anisotropy metric is calculated from the same DWIs used for the MD estimation.

On the other hand, the use of 6 gradient directions to better characterize diffusion is not only related with the 6 degrees of freedom of the diffusion tensor. It is well-known that there is an intrinsic limitation in dMRI that hinders the proper estimation of anisotropy measures to characterize fibers in all different spatial directions. This effect also affects the method here proposed, imposing a limitation of use. We have shown that those fibers that are not aligned with the axes will be underestimated, the larger the misalignment the larger the underestimation. Thus, this method is not able to circumvent this intrinsic limitation of dMRI and therefore it must be used with caution. The purpose of the method is not to be used in clinical studies or as a substitute of the FA, but to provide complimentary information in fast diffusion acquisitions. There is a clear loss of information when compared with a complete dMRI acquisition, but there is also a clear additional information when compared with that provided only by the MD. In this sense, the advantage of using DiA is its ability to provide anisotropy information with the smallest possible data set.

## Footnotes

1 Data obtained from the Human Connectome Project (HCP) database (ida.loni.usc.edu/login.jsp). The HCP project (Principal Investigators: Bruce Rosen, M.D., Ph.D., Martinos Center at Massachusetts General Hospital; Arthur W. Toga, Ph.D., University of Southern California, Van J. Weeden, MD, Martinos Center at Massachusetts General Hospital) is supported by the National Institute of Dental and Craniofacial Research (NIDCR), the National Institute of Mental Health (NIMH) and the National Institute of Neurological Disorders and Stroke (NINDS). HCP is the result of efforts of co-investigators from the University of Southern California, Martinos Center for Biomedical Imaging at Massachusetts General Hospital (MGH), Washington University, and the University of Minnesota.

2 A “uniform” downsampling of *n* gradients among the original 64 is here defined as those *n* directions that minimize the overall electrostatic repulsion energy among all $\left(\begin{array}{c} 64\\ n \end{array}\right)$ combinations. The optimization is carried out using heuristic rules.
